# Historical Introgression of the Downy Mildew Resistance Gene *Rpv12* from the Asian Species *Vitis amurensis* into Grapevine Varieties

**DOI:** 10.1371/journal.pone.0061228

**Published:** 2013-04-12

**Authors:** Silvia Venuti, Dario Copetti, Serena Foria, Luigi Falginella, Sarolta Hoffmann, Diana Bellin, Petar Cindrić, Pál Kozma, Simone Scalabrin, Michele Morgante, Raffaele Testolin, Gabriele Di Gaspero

**Affiliations:** 1 Dipartimento di Scienze Agrarie e Ambientali, University of Udine, Udine, Italy; 2 Istituto di Genomica Applicata, Parco Scientifico e Tecnologico Luigi Danieli, Udine, Italy; 3 Research Institute of Viticulture and Enology, University of Pécs, Pécs, Hungary; 4 Faculty of Agriculture, University of Novi Sad, Novi Sad, Serbia; Kansas State University, United States of America

## Abstract

The Amur grape (*Vitis amurensis* Rupr.) thrives naturally in cool climates of Northeast Asia. Resistance against the introduced pathogen *Plasmopara viticola* is common among wild ecotypes that were propagated from Manchuria into Chinese vineyards or collected by Soviet botanists in Siberia, and used for the introgression of resistance into wine grapes (*Vitis vinifera* L.). A QTL analysis revealed a dominant gene *Rpv12* that explained 79% of the phenotypic variance for downy mildew resistance and was inherited independently of other resistance genes. A Mendelian component of resistance–a hypersensitive response in leaves challenged with *P. viticola*–was mapped in an interval of 0.2 cM containing an array of coiled-coil NB-LRR genes on chromosome 14. We sequenced 10-kb genic regions in the *Rpv12^+^* haplotype and identified polymorphisms in 12 varieties of *V. vinifera* using next-generation sequencing. The combination of two SNPs in single-copy genes flanking the NB-LRR cluster distinguished the resistant haplotype from all others found in 200 accessions of *V. vinifera*, *V. amurensis*, and *V. amurensis* x *V. vinifera* crosses. The *Rpv12^+^* haplotype is shared by 15 varieties, the most ancestral of which are the century-old ‘Zarja severa’ and ‘Michurinets’. Before this knowledge, the chromosome segment around *Rpv12^+^* became introgressed, shortened, and pyramided with another downy mildew resistance gene from North American grapevines (*Rpv3*) only by phenotypic selection. *Rpv12^+^* has an additive effect with *Rpv3^+^* to protect vines against natural infections, and confers foliar resistance to strains that are virulent on *Rpv3^+^* plants.

## Introduction

The Amur grape (*Vitis amurensis* Rupr.) is native to cool climates of the Far East, including Siberia, China, Korea, and Japan [1]. *V. amurensis* is a dioecious species. A few forms of what is believed to be pure *V. amurensis* with domestication traits–i.e. relatively large berries and hermaphroditic flowers–are grown in northeastern China [2] or reported in literature [3], [4]. This species is a reservoir of important viticultural traits that are absent from the germplasm of the European wine grape *Vitis vinifera*. The Amur grape is winter hardy–an adaptive trait to withstand temperatures as low as −40°C in its natural habitat and a long-sought character for cool climate viticulture. Some accessions are not significantly damaged by *Plasmopara viticola* under conditions highly conducive to downy mildew [5]. That is why the species has attracted grape breeders' attention since the collecting expeditions in the Far East (1920–1940) organised by the Russian botanist Vavilov [6]. Hybridisation with introduced accessions of *V. amurensis* became the method of choice to strengthen cold hardiness and downy mildew resistance in *V. vinifera*. Michurin, Negrul, and Potapenko are credited with the first use of *V. amurensis* for grape breeding in continental climates. Intentional crosses with *V. amurensis* also started in China after World War II [7]. Until recently, grape breeding in the former Soviet Union and China was isolated from the influence of the Western world, where grape improvement for disease resistance has employed only North American genetic resources for almost a century. A few North American founders gave rise to large descent groups of resistant breeding lines. Several haplotypes of the most documented North American resistance gene for downy mildew (*Rpv3*) have been introgressed since the mid 1800s into many varieties [8]. *Rpv3* is located on chromosome (chr) 18 [9] and confers a race-specific hypersensitive response (HR) against *P. viticola* strains that carry the cognate avirulence factor [10]. French-American hybrids carrying the *Rpv3* gene have become popular in all European countries committed to grape breeding. Thanks to the networking in the former Soviet bloc, grape breeders in Eastern Europe took advantage of sharing *V. amurensis* germplasm with the Soviet centres of Michurinsk, Novocherkassk, and Magarach, and since the 1960s this material was introduced into continental Europe [11]. *Rpv10* is the first mapped resistance gene against downy mildew that was introgressed from *V. amurensis* into grapevine varieties of Western Europe [12]. Many resistant varieties such as Severnyi, Rondo, and Solaris–selected in Russia, Czechoslovakia, and Germany–share *Rpv10* by descent [12] but many others are derived from *V. amurensis* and show high levels of resistance in the absence of known resistance genes.

In this paper, we report on another resistance gene donated by *V. amurensis* to existing grape varieties. The locus has been named *Rpv12* and listed ahead of publication in the catalogue of mapped resistance loci, following the guidelines of the International Grape Genome Program. *Rpv12* is inherited independently of other resistance genes introgressed in cultivated grapevines and has become fixed in the genetic background of several varieties, alone or in combination with a resistant haplotype of the gene *Rpv3*.

## Materials and Methods

### Plant material and P. viticola strains for phenotyping

Pedigrees of custom-made populations and varieties derived from *V. amurensis* are reported in Figure S1. *Rpv3* and *Rpv10* haplotypes were determined using markers reported in [8] and [12]. Varieties and wild accessions used for genotyping at the *Rpv12* locus are listed in Figure S2. Accessions of unknown origin were also genotyped with unlinked microsatellite markers (Figure S3).

Phenotyping of mapping populations was done using biological replicates raised from rooted cuttings taken from seedlings. In field experiments, vineyards were maintained following standard crop practices and withholding the application of fungicides with activity against *P. viticola*. Resistance was scored during two consecutive seasons using the OIV452 descriptor. For leaf disc assays, grapevines were grown in a greenhouse prior to inoculation. QTL analysis was performed upon inoculation with the NE-I isolate of *P. viticola* [9]. Phenotyping of recombinant individuals was extended to two isolates of *P. viticola* characterised by [10]. Sixteen leaf discs were excised from the fourth and fifth leaves beneath the shoot apex of two clonal replicates and plated onto wet paper with the abaxial side up. Discs were sprayed with a suspension of *P. viticola* at 150,000 sporangia ml^−1^ and incubated at 21°C and day length of 16 h, for 8 days. Presence/absence of HR and extent of pathogen sporulation were scored as described in [9]. Briefly, the OIV452 scale is positively correlated with the magnitude of plant response and inversely correlated with the severity of downy mildew symptoms. A cumulative rating (∑OIV452) was obtained by summing the scores from 3 to 8 days post inoculation (dpi). Field experiments were conducted at the Experiment Farm ‘A. Servadei’ owned by the University of Udine, Italy. The land accessed is not a protected area. No protected or endangered plant species were sampled. All necessary permits for the described field studies were issued by the Regional Government of Friuli Venezia Giulia, Italy, Authorisation no. D.P.Reg.n.0198/Pres.2004 no. 322/2007.

### QTL analysis

Microsatellite and RGA markers were mapped as described in [13]. Parental genetic maps were constructed with JoinMap 4.0. Linkage groups were built at an LOD threshold of 4.0, and chromosome number was assigned according to [14]. QTL analysis was performed by interval mapping using MapQTL 5.0. The LOD threshold for significant QTLs at α = 0.05 was estimated using a permutation test with 1,000 iterations. The confidence interval was initially defined as a one-LOD support interval and then refined using informative recombinants with extreme phenotypes for ∑OIV452 supported by scores of the HR.

### Microsatellite and SNP development, genotyping, and haplotype reconstruction

Microsatellite and SNP markers were designed in the upper arm of chr14, and used for linkage mapping and haplotyping (Figure S4). SSR amplicons were sized using an AB3730 capillary sequencer and alleles were called with GeneMapper v4.0 (Applied Biosystems). For SNP discovery and mapping, primers were designed in exons of PN40024 single-copy genes. Amplicons were captured by Agencourt AMPureXP (Beckman Coulter) magnetic beads and used for Sanger sequencing of both DNA strands. Sanger reads were trimmed using Phred and consensus sequences were created using Phrap. Nucleotides were referenced to the chromosomal coordinates of the PN40024 12X assembly by BlastN alignments. For SNP haplotyping, a total of ∼3 billion Illumina 100-bp paired-end reads were obtained from nuclear DNA of twelve grapevine varieties (Aglianico, Barbera, Cannonau, Carignan, Kishmish vatkana, Nebbiolo, Pinot noir, PN40024, Primitivo, Sangiovese, Schiava grossa, Vermentino), quality trimmed, and filtered for contaminants and duplicates. Paired reads were aligned with Burrows-Wheeler Alignment (BWA) to the *Rpv12^+^* consensus sequence. Raw sequencing data are deposited in the Sequence Read Archive (SRP018751). Variants were called with VarScan.v2.2.8 using uniquely mapped reads. Haplotypes were determined using PHASE, guided by the evidence of neighbouring phased variants present within a read or in paired reads. Cladograms were constructed using the UPGMA method. The most informative SNPs were validated by PCR amplification and Sanger sequencing in an extended set of grapevine varieties (Figure S1–S2). Measures of DNA sequence variation were estimated using DnaSP.

## Results

### QTL mapping of Rpv12

QTL analysis was conducted in two populations of collectively 180 offspring. One population was created by crossing a heterozygous line (*Rpv12^+^*/*Rpv12^−^*), in which *Rpv12* was the only segregating resistance gene, with a sensitive grapevine (*Rpv12^−^*/*Rpv12^−^*). The genetic map of the *Rpv12^+^*/*Rpv12^−^* line consisted of 280 markers (Figure S5A). Another population was a dihybrid cross between a double heterozygous resistant parent (*Rpv12^+^*/*Rpv12^−^*, *Rpv3^+^*/*Rpv3^−^*), in which a known resistance gene (*Rpv3*, [9]) was segregating along with *Rpv12* in the same parent, and a double homozygous recessive grapevine (*Rpv3^−^*/*Rpv3^−^*, *Rpv12^−^*/*Rpv12^−^*). The genetic map of the *Rpv3^+^*/*Rpv3^−^*, *Rpv12^+^*/*Rpv12^−^* line consisted of 175 markers (Figure S5B).

**Figure 1:**
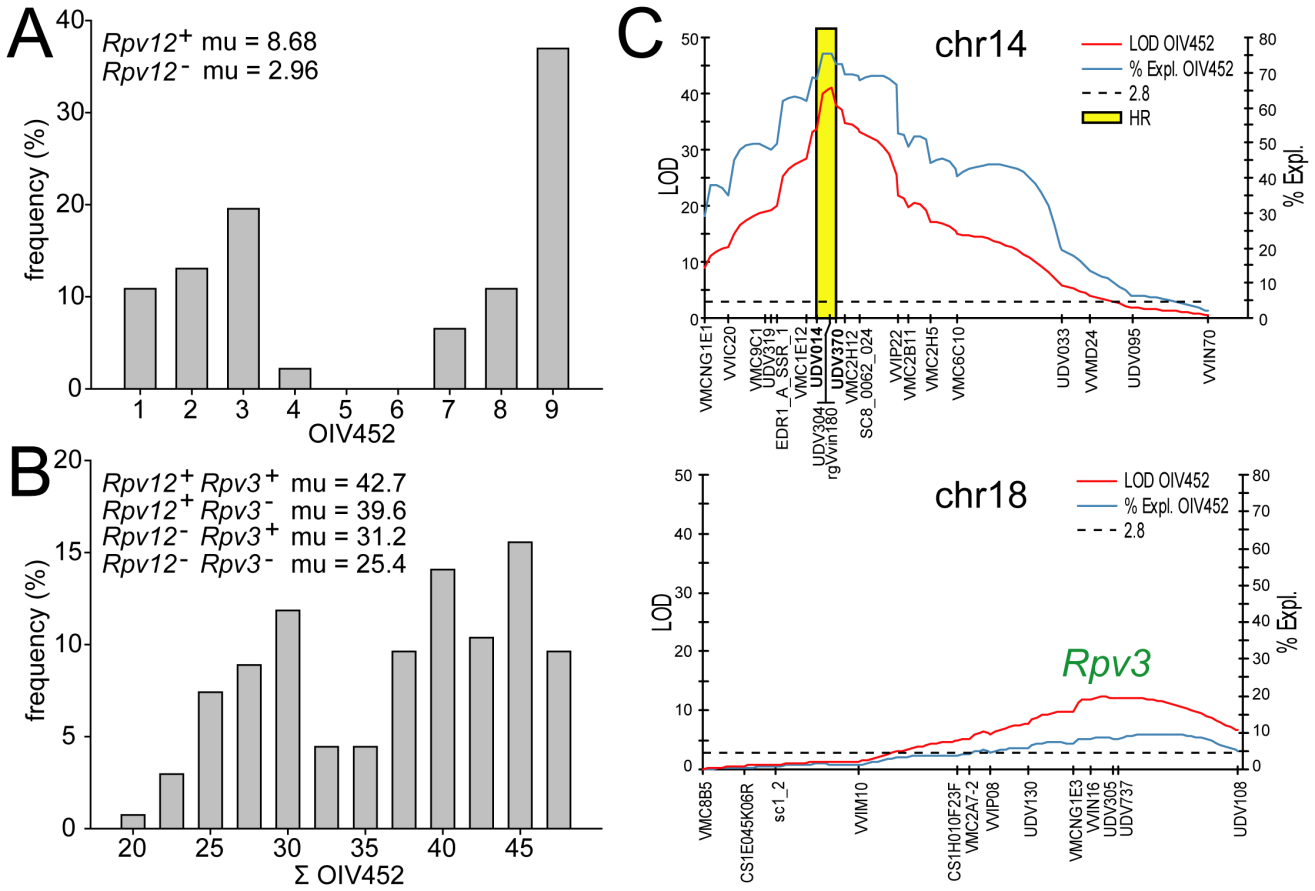
Phenotypic distribution of downy mildew resistance. Two families segregating for *Rpv12^+^* (panel **A**) and for the combination of *Rpv12^+^* and *Rpv3^+^* (panel **B**) were analysed. Resistance scores in panel **A** are based on the OIV452 parameter (1 = most sensitive, 9 = most resistant) scored on field-grown seedlings under natural infection. Resistance scores in panel **B** are based on the cumulative OIV452 parameter (∑OIV452 = sum of daily OIV452 scores from 3 to 8 dpi) in artificially inoculated leaf discs. The average phenotypic value in the upper left corner of the panels **A**–**B** refers to individuals grouped by their allelic status at the *Rpv12* and *Rpv3* genes, which was estimated based on the flanking markers UDV014/UDV370 for *Rpv12*, and on UDV305/UDV737 for *Rpv3* [8]. Recombinants in those intervals were excluded from this estimate. QTL plots that explain the phenotypic variance shown in panel **B** are given in panel **C**.

DM resistance segregated in a bimodal fashion in the progeny of the *Rpv12^+^*/*Rpv12^−^*, *Rpv3^−^*/*Rpv3^−^* parent (Figure 1A). The *Rpv12^+^* locus was located on chr14 and explained 78.7% of the phenotypic variance for the OIV452 parameter (LOD = 15.4) in field-grown grapevines exposed to natural infection (Figure S5C). The QTL peak was associated with markers UDV014 and UDV370. In the progeny, the presence of the UDV014 and UDV370 alleles in coupling with *Rpv12^+^* was associated with an average phenotypic value of 8.68 for the OIV452 parameter, their concomitant absence with a value of 2.96.

QTL analysis in the progeny of the double heterozygous resistant parent (*Rpv12^+^*/*Rpv12^−^*, *Rpv3^+^*/*Rpv3^−^*) confirmed the location of the QTL peak in the interval delimited by markers UDV014 and UDV370, and the independent assortment of *Rpv12* and *Rpv3* alleles (Figure S5D). In the leaf disc assay, *Rpv12* explained 74.5% of the phenotypic variance for the ∑OIV452 parameter (LOD = 41), while 8.7% of the residual variance was accounted for by a QTL peaking between markers UDV305 and UDV737 on chr18 (LOD = 12.4), which corresponded to the *Rpv3* locus (Figure 1B–C). Maximum LOD for the *Rpv12* locus was associated with the markers rgVvin180 and UDV304 previously designed in NB-LRR genes [15], which were located within the one-LOD interval delimited by markers UDV014 and UDV370. *Rpv12* had a stronger effect than *Rpv3* on the ability of the seedlings to restrict pathogen sporulation, and the two loci had an additive effect. Compared to sensitive double homozygous recessive seedlings that scored an average ∑OIV452 value of 25.4, *Rpv3^+^* seedlings scored 31.2 (+23%), which rose to 39.6 (+56%) in *Rpv12^+^* seedlings. The combination of both resistance genes in *Rpv12^+^ Rpv3^+^* seedlings was associated with the highest ∑OIV452 value of 42.7 (+68%).

### The Rpv12 phenotype

**Figure 2:**
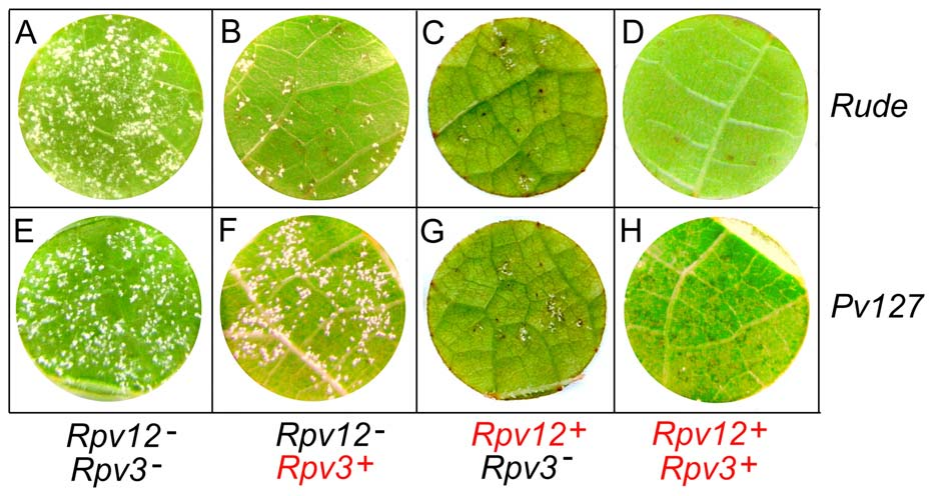
Host–pathogen interaction observed between host and pathogen genotypes. Leaf discs of four host genotypes including (panels **A**, **E**) a double homozygous recessive grapevine (*Rpv12^−^* and *Rpv3^−^*), (panels **B**, **F**) a grapevine carrying *Rpv3^+^* in the absence of *Rpv12^+^*, (panels **C**, **G**) a grapevine carrying *Rpv12^+^* in the absence of *Rpv3^+^*, and (panels **D**, **H**) a double heterozygous grapevine for both *Rpv12^+^* and *Rpv3^+^* were inoculated with two isolates of *P. viticola*, (panels **A**–**D**) *Rude* (*avrRpv3^+^*/*avrRpv12^+^*) and (panels **E**–**H**) *Pv127* (*avrRpv3^−^*/*avrRpv12^+^*). Pictures were taken at 6 dpi.

*Rpv12^+^* conferred the ability to establish an HR within 24–48 hours post inoculation and to restrict sporulation of *P. viticola* (Figure 2C, G). The type and the timing of the plant reaction are similar to those triggered by *Rpv3* [9], but the limitation imposed on pathogen sporulation is more significant (Figure 1B, Figure 2B, C). *Rpv12*-dependent HR was successfully established in response to at least one isolate of *P. viticola* that eludes or suppresses the resistance conferred by *Rpv3* (Figure 2F, G). The isolate *Pv127* that equally sporulated on *Rpv3^−^* and *Rpv3^+^* hosts (Figure 2E, F and [10]) was halted on *Rpv12^+^* grapevines to the same extent as the other isolate (Figure 2). The combination of the resistance alleles *Rpv12^+^* and *Rpv3^+^* restricted pathogen growth to the highest extent (Figure 1B), regardless of the pathogen strain (Figure 2D, H).

### Mendelisation and fine mapping of Rpv12

The genetic interval underlying the QTL peak for DM resistance was delimited between markers UDV014 and UDV370, based on the assignment of each offspring as being either able or unable to establish the HR (Figure 1A). Validation of this interval was performed in a distinct family of 185 seedlings created by crossing a sensitive grapevine with one of the earliest available ancestors of the *Rpv12^+^* lineage–the variety Kunbaràt (Figure S6). Fine mapping of *Rpv12^+^* was initiated from the markers UDV014 and UDV370, which were used for DNA genotyping of 2,532 individuals segregating for *Rpv12^+^* (Figure S1). The occurrence of 16 recombinants in the interval (0.6 cM) allowed us to dissect the locus by the targeted design of microsatellite and SNP markers (Figure S7A). To this end, we amplified 14 fragments in 8 single-copy genes from genomic DNA of an *Rpv12^+^* homozygous line (hereafter called ‘156,623’) and sequenced 10,675 nucleotides of the *Rpv12^+^* haplotype (Figure S4). The *Rpv12^+^* haplotype was compared with 23 haplotypes identified in 12 varieties of *V. vinifera*. The nucleotide diversity π value was 0.00313 in the sample of *V. vinifera*, and the number of net nucleotide substitutions per site was 0.00668 between the *Rpv12^+^* haplotype and *V. vinifera* haplotypes. All variable sites between *Rpv12^+^* and *V. vinifera* haplotypes are indicated in Figure S7B-C, and those variants uniquely found in *Rpv12^+^* are highlighted.

Informative SNPs were mapped on the recombinant chromosomes available to this study and reduced the genetic interval of *Rpv12^+^* to a 0.24-cM region spanning a cluster of coiled-coil (CC)-NB-LRR genes (Figure S7A). The borders of the locus are defined upstream by marker SNP 14_ 9036110 (chr14:9.036 Mbp) in the gene *pentatricopeptide repeat-containing protein* (GSVIVT00036207001) and downstream by marker SNP 14_ 9882587 (chr14:9.882 Mbp) in the gene *serine*/*threonine protein phosphatase* (GSVIVT00023546001). Another five genes (peptidyl-prolyl cis-trans isomerase CYP19-3, bHLH104-like transcription factor, AP2/ERF domain-containing transcription factor, hypothetical protein coding region, and activating signal cointegrator) are located between the upper edge of the NB-LRR cluster and the upper border of the locus. *Rpv12^+^* is removed from the upstream SNP 14_ 9036110 by 2 events of crossing over in 2,532 meioses and by 4 crossovers from the downstream marker SNP 14_ 9882587. The resistant haplotype in the homozygous line ‘156,623’ has cytosines at both SNP 14_ 9036110 and SNP 14_ 9882587 positions, which define the CC haplotype of the resistant allele *Rpv12^+^* and distinguish it from the TA haplotype present in all accessions of *V. vinifera* tested (Figure S2). The C variant at either marker is also present in wild accessions of *V. amurensis* and *V. coignetiae*, but we did not find the CC haplotype in any of the *V. amurensis* breeding lines that do not share *Rpv12^+^* by descent, i.e. ‘Severnyi’, ‘Beichun’, and ‘Michurinski’ (Figure S2). The *V. vinifera* haplotype TA is ancestral to the *V. amurensis* haplotype CC because the haplotype TA was also found in other Asian and North American species (Figure S7D). For DNA tests in controlled-cross progeny, these universal SNP markers can be replaced without substantial increase in recombination frequency by nearby microsatellite markers, which reveal allelic variants by size and are heterozygous in *Rpv12^+^* varieties (Figure S8L). On the upper side of *Rpv12*, UDV343 and UDV345 cosegregate with SNP 14_ 9036110 while UDV350 is placed 0.08 cM outwards; on the lower side, UDV360 is 0.04 cM outward from SNP 14_ 9882587. Another six microsatellite markers (UDV340, UDV347, UDV348, UDV349, UDV380, and UDV390) cosegregate with the *Rpv12* phenotype as well as marker SNP 14_ 9036110 (chr14:9.257 Mbp), which is located in the single-copy gene (*Activating signal* cointegrator, GSVIVT00036192001) next to the upper edge of the NB-LRR cluster.

The number of net nucleotide substitutions per site between *Rpv12^+^* and *V. vinifera* haploypes decreased from each flanking region towards the *Rpv12^+^* NB-LRR gene cluster (Figure S7D). We also detected a reduction of haplotype diversity, which was confirmed by an extended analysis in other *Vitis* species. In the *activating signal cointegrator* gene, we failed to find any allelic variant that distinguished the *Rpv12^+^* haplotype from all varieties of *V. vinifera*, while diversity was detected within the population of *V. vinifera* and among accessions of other Asian and North American species (i.e. *V. yenshanensis*, *V. monticola*, *V. cordifolia*, Figure S7B).

### Origin of the Rpv12^+^ haplotype and linkage drag in introgression lines

The *Rpv12^+^* haplotype donated by the original individual of *V. amurensis* was inferred from the comparison of the most ancient varieties ‘Zarja severa’ and ‘Michurinets’ that are heterozygous for *Rpv12^+^*/*Rpv12^−^* and were selected from the first filial generation of pure *V. amurensis* x *V. vinifera* crosses (Figure S8G). Phasing of marker alleles in the *Rpv12^+^* haplotype was determined using the seedling ‘156,623’, which carries *Rpv12^+^* in a homozygous state from marker sc30_4 (chr14:4.025 Mbp) to VMC2H5 (chr14:20.981 Mbp), and the variety *V. vinifera* ‘Italia’, because it shares with ‘Michurinets’ the *V. vinifera* haplotype from marker VVIC20 (chr14:3.125 Mbp) to marker VVIP22 (chr14:18.364 Mbp). This analysis also included the control DNA samples of ‘PN40024’ and its ancestor ‘Schiava Grossa’, which donated the chromosome fragment containing the *Rpv12^−^* haplotype to ‘PN40024’ (see [14] for the pedigree of ‘PN40024’).

The extent of linkage drag around *Rpv12^+^* was estimated in 21 varieties phenotypically selected from different generations of backcross, and compared to F1 hybrids (Figure S1, S8L). In ‘Zarja severa’, ‘Michurinets’, and ‘156,623’, the linkage around *Rpv12^+^* persisted until marker VMC1E12 (chr14:7.137 Mbp) upstream of *Rpv12^+^* and until marker VMC2H5 (chr14: 20.981 Mbp) downstream of *Rpv12^+^*. In varieties further removed from the wild parent, linkage drag was eliminated in ‘13-12-15’ immediately downstream of *Rpv12^+^* in the *serine/threonine protein phosphatase* gene next to the NB-LRR cluster (chr14:9.882 Mbp Figure S2), which was confirmed by the alleles at the nearby marker UDV360 (chr14:9.910 Mbp, Figure S8L). ‘Bruskam’, ‘Kunleany’, and ‘Taurus’ also have minimal linkage drag downstream of *Rpv12^+^*. On the other side of the *Rpv12* locus, ‘Stepnyak’ had the least linkage drag of wild alleles in coupling with *Rpv12^+^*, which persisted for 1.4 Mbp away from the upper edge of the NB-LRR cluster but were not present from VMC1E12 to the top of chr14. With the exception of ‘Stepnyak’, the varieties that share *Rpv12^+^* are all identical to one another and with the hybrids ‘Zarja severa’ and ‘Michurinets’ in the chromosomal region comprised between VMC1E12 and UDV340, but they differ upstream in completely or partially sharing a haplotype with either ‘Zarja severa’ or ‘Michurinets’.

In a set of 48 accessions of *V. amurensis* and early descendents of *V. amurensis* x *V. vinifera* crosses conserved in breeding centres, we did not find any DNA profile to be compatible with the parent that donated *Rpv12^+^* to ‘Zarja severa’ or ‘Michurinets’ (Figure S4). We were also unable to find an offspring or a full/half-sibling of ‘Zarja severa’ or ‘Michurinets’ among the selections that carry *Rpv12^+^* (Figure S4 and S9). All resistant varieties that contain *Rpv12^+^* tested negative for the resistant haplotype *Rpv10^+^* transmitted by ‘Severnyi’ and *vice versa* (Figure S1). However, some *Rpv12^+^* varieties did test positive for the resistant haplotype of *Rpv3* transmitted by ‘Seibel 4614’ (Figure S1). Before this molecular characterisation of *Rpv12^+^* donors, two resistant descendents of *V. amurensis* (‘13-12-17’ and ‘13-12-15’) were crossed for breeding purposes and the ten most resistant offspring were retained (Figure S1, family G). Since ‘13-12-15’ was the only parent to contain *Rpv12^+^*, we genotyped all phenotypically selected offspring and assessed that all contained *Rpv12^+^*.

### NB-LRR genes in the Rpv12 locus and in the upper arm of chromosome 14

There are 13 CC-NB-LRR genes in the *Rpv12* locus of the reference grapevine genome with a high degree of sequence identity (Figure S7F). This array of NB-LRRs spans ∼600 kb and it is interspersed with another three genes with unrelated functions (asparagine synthetase B, cytochrome P450 89A2, and a hypothetical protein coding region). The *Rpv12* NB-LRR array is part of a more complex structure of 46 clustered NB-LRRs in the upper arm of chr14 (Figure S8N). All NB-LRRs are of CC-type and are physically located in the uppermost 14 Mbp of the chromosome. The pattern of repetitive elements and recombination rate consistently predict the location of the centromere at ∼15 Mbp between markers SC8_0062_ 024 and VVIP22 (Figure S8A,C). All but one NB-LRR on chr14 arose from intrachromosomal duplications and form a clade that roots deep in the cladogram of the NB-LRR gene family (Figure S8M,N,P). In addition to the 13 CC-NB-LRR genes in the *Rpv12* locus, which derived from short-range duplications, other CC-NB-LRR genes are organised into another four clusters, with the exception of an isolated copy at 14.6 Mbp that is of CC-type but unrelated with respect to the others (Figure S8O). Outside of the *Rpv12* locus, the closest NB-LRR genes are arrayed ∼3 Mbp away towards the upper telomeric end, and ∼2 Mbp away towards the centromere.

### Estimation of the sequence gap in the Rpv12 locus of the reference grapevine genome

The assembly of the grapevine reference genome has a scaffold junction of unknown size within the *Rpv12* locus due to a high degree of duplication that hampered scaffolding of the arrayed NB-LRR genes. The NB-LRR sequences in the *Rpv12* locus have all of their reciprocal best matches in the contiguous terminal ends of scaffolds 81 and 36. We did not find highly similar paralogues in unanchored scaffolds, easing the concern that the gene array in PN40024 may contain more locally duplicated copies.

In order to estimate the size of the physical gap, we used the physical contig_1449 of the ‘Cabernet Sauvignon’ BAC library, which tested positive for the NB-LRR marker UDV304 [15] that was genetically mapped to *Rpv12* (Figure S7E) but lacked any match of its primer sequences in the PN40024 reference sequence. We selected all unique end sequences of the BAC clones included in contig_1449 and projected them onto the PN40024 assembly by BlastN search. All sequences that passed the thresholds of >50% sequence aligned and >90% sequence identity matched the contiguous terminal ends of scaffolds 81 and 36. In particular, BAC_33J08 spanned the scaffold junction. One end of BAC_68L04–the mate of the sequence from which marker UDV304 was developed–matched sequences in scaffold 81, while the other end was repetitive within the *Rpv12* NB-LRR cluster.

## Discussion

The *Rpv12* locus coincides with a cluster of CC-NB-LRR genes on chromosome 14 and the inheritance of the resistant haplotype is associated with a localised HR in leaves challenged with *P. viticola*. *Rpv12* adds to the list of functional NB-LRR genes introgressed into the *V. vinifera* genome for improving downy mildew resistance, now including *Rpv1*, *Rpv3*, *Rpv10*, and *Rpv12* [9], [12], [16]–the latter two being donated by accessions of *V. amurensis*. *Rpv12^+^* plants are able to mount a type of host resistance similar to that observed in North American grapevines, raising the intriguing question as to how the functionality of *Rpv12* against *P. viticola* has evolved in the Asian *V. amurensis* without co-evolution and in the absence of the pathogen.

Compared to the loci *Rpv1* and *Rpv3*, which contain TIR-NB-LRR genes, *Rpv12* contains exclusively CC-NB-LRR genes, a typically less dynamic subclass. According to [17], TIR-NB-LRR genes undergo sequence exchange at higher rates and with greater functional constraints to keep pace with changes in the population of specialised pathogens, while CC-NB-LRR genes are evolutionary more stable, preserving established functionalities against a wider range of less-adapted biotic threats. The presence in the Asian continent of *Plasmopara cissii* and *Plasmopara amurensis* that are adapted to other Vitaceae species [18] suggests that the *Rpv12* locus of the genus *Vitis* contains functional remnants against *Plasmopara* species with loose recognition specificity. In our experiments, *Rpv12^+^* protects grapevines from the threat of downy mildew more effectively than the North American gene *Rpv3*, by expanding the recognition spectrum to isolates of the pathogen that are virulent on *Rpv3^+^* plants.

*Rpv12* is inherited independently of another resistance gene *Rpv10* introgressed from *V. amurensis* into varieties of *V. vinifera* [12]. DNA tests provided strong support for the hypothesis that ‘Zarja severa’, which contains *Rpv12^+^*, and ‘Severnyi’, which contains *Rpv10^+^*, are full-siblings. According to historical records, the cross that gave rise to ‘Zarja severa’ and ‘Severnyi’ was made in 1936 by Potapenko and Zakharova at the Michurin Central Genetics Laboratory in Michurinsk, Russia. The reported direction of the cross indicates that *V. amurensis* was the pollen parent and, if purely wild, it should have been a staminate plant. Under this hypothesis, *Rpv12^+^* and *Rpv10^+^* were donated by the same male accession of *V. amurensis*, and they segregated in the selected offspring. Another possible direct descendent of the same resistant accession of *V. amurensis* is ‘Michurinets’, which has a coefficient of relatedness compatible with sharing one parent with ‘Zarja severa’ and ‘Severnyi’, and contains *Rpv12^+^* but not *Rpv10^+^*. None of the other varieties that were reported to originate from the first filial generation of *V. amurensis* and contain *Rpv12^+^* (‘Bruskam’, ‘13-12-15’, and ‘13-12-11/K10’) are full-siblings or half-siblings with ‘Zarja severa’ and ‘Michurinets’ (Figure S9), falsifying the hypothesis that they have a parent-offspring relationship with the same wild accession that donated *Rpv12^+^* to ‘Zarja severa’ and ‘Michurinets’. In the set of breeding lines available to this study, we identified 28 varieties and breeding lines that contain *Rpv12^+^*, and another 12 that contain *Rpv10^+^*, but we did not find any variety with both *Rpv10^+^* and *Rpv12^+^*. It is curious that seedlings containing only one of the two genes were accidentally selected–despite the expectation that seedlings retaining both genes are phenotypically superior–and that the genes did not become combined again by crossing lineages that descended from ‘Zarja severa/Michurinets’ and ‘Severnyi’, despite their diffusion in many breeding centres. In contrast, empirical evidence has guided breeders to cross grapevines that we now know to carry the *V. amurensis Rpv12^+^* haplotype with French-American lines of the ‘Seibel 4614’ lineage that we now know to carry an *Rpv3^+^* resistant haplotype. Pyramidisation of resistance genes was realised before the advent of DNA testing by the phenotypic selection of the best parents to cross and the most resistant seedlings to retain (Figure S1). The resistance phenotype in *Rpv12^+^/Rpv3^+^* grapevines was fortified by the combination of two loci that contain CC-NB-LRR and TIR-NB-LRR genes.

An additional 10 descendents of *V. amurensis* x *V. vinifera* crosses have neither *Rpv12^+^* nor *Rpv10^+^* (Figure S1). There is evidence to suggest that some of them (‘Beichun’, ‘Bashkanskii krasnyi’, data not shown) are able to significantly restrict the sporulation of downy mildew, which leads to the assumption that other resistant haplotypes/genes have been introgressed from *V. amurensis*.

In this study, the resistant phenotype conferred by *Rpv12^+^* was always dependent on an HR and significant limitation–but not complete absence–of pathogen sporulation in different genetic backgrounds. This phenotype differs from the complete resistance claimed in literature reports for another resistance locus identified in wild *V. amurensis* (*Rpv8*, [4]). These phenotypic observations weaken the hypothesis that *Rpv8* and *Rpv12* resistance specificities are conferred by the same haplotype, prompting us to name the haplotype analysed in this paper separately from *Rpv8*. The low resolution of the QTL plot of *Rpv8* described in [4] does not allow the exclusion of the possibility that *Rpv12* and *Rpv8* are either alleles in the germplasm of *V. amurensis* or paralogous loci. In the upper arm of chr14 of the reference grapevine genome, there are 48 NB-LRR genes, pseudogenes, and gene fragments, 13 of which are physically located in the genetic interval of *Rpv12*. Another two major clusters are located ∼2 Mbp and ∼3 Mbp away.

The *V. amurensis Rpv12^+^* haplotype is highly diverse with respect to *V. vinifera* haplotypes (Figure S7), but nucleotide diversity was lower in the single-copy genes most proximal to both sides of the *Rpv12^+^* NB-LRR cluster than in more distant genes. In order to assess whether nucleotide diversity in the *Rpv12* locus is also reduced in other *Vitis* accessions, we sequenced the GSVIVT00036207001 gene fragment in all wild accessions available to this study. The haplotypes detected in the single-copy gene next to the upper edge of the NB-LRR cluster conflicted with the phylogeny, as we observed more differentiation within species (i.e. among varieties of *V. vinifera*) than between Eastern Asian–Eastern North American disjuncts, and more differentiation between haplotypes of a single diploid accession than between haplotypes of different species (Figure S7D). It is possible that this inconsistency is due to the amount of incomplete lineage sorting–the stochastic retention of ancestral polymorphisms in recently diverged species [19]. Divergence among *Vitis* species has in fact occurred in the past 6 million years [20] and lineages in *V. vinifera* have larger widths than lengths [21], in the absence of significant bottlenecks during the recent history of domestication and cultivation [22].

By analysing varieties and new seedlings that carry *Rpv12^+^*, we showed that recombination is not suppressed at the chromosomal scale between the *V. amurensis* haplotype *Rpv12^+^* and haplotypes in *V. vinifera*. During the process of introgression into the *V. vinifera* background, linkage drag could be minimised on each side of the *Rpv12* NB-LRR gene cluster. In the past, without the assistance of DNA typing, the introgressed segment around *Rpv12^+^* has been shortened in backcross generations, and the varieties ‘13-12-15’ and ‘Stepnyak’ are those with the least linkage drag. For practical breeding, we demonstrated that screening ∼2,500 new seedlings was sufficient to identify four recombinants at the very lower edge of the NB-LRR gene cluster and, on the other side, two recombinants that retained only five genes of the resistant haplotype past the NB-LRR gene cluster. We expect that linkage drag around the NB-LRR gene cluster will be eliminated by backcrossing these individuals to *V. vinifera* and raising a few thousand seedlings.

## Conclusions

A downy mildew resistance gene of the Asian species *V. amurensis* was initially introgressed by Soviet breeders into *V. vinifera* and then combined by European breeders with other resistance genes from North American grapevines. Our molecular analyses showed that grape breeders over the past century were visionary in the choice of the parents to cross, the seedlings to retain, and the resistant lines to combine. Marker assisted selection is now feasible with the DNA information made available by this paper. We showed that the haplotype analysis of the introgressed segment provides plenty of markers to track the gene and guide the elimination of linkage drag around the introgressed gene. Background selection is key to the quality of the new grape varieties created by conventional breeding.

## Supporting Information

Figure S1 Pedigrees of custom-made populations for genetic mapping, and varieties containing the resistant haplotype of *Rpv12*.
(JPG)

Figure S2 List of 200 grapevine accessions and their genotypes at the *Rpv12* locus. DNA typing was performed by Sanger sequencing of the gene fragments GSVIVT00036207001 and GSVIVT00023546001 and by scoring nucleotide variants at the chromosomal positions chr14:9036110 and chr14: 9882587, flanking the *Rpv12* NB-LRR cluster.
(JPG)

Figure S3 DNA fingerprinting and kinship of 48 accessions of *V. amurensis*, descendents of *V. amurensis* x *V. vinifera* crosses with unknown pedigree, and introgression lines in the first two generations of descent with known pedigree.
(JPG)

Figure S4 Microsatellite and SNP markers on chr14.
(JPG)

Figure S5 Genetic maps and QTL plots for downy mildew foliar resistance (OIV452 parameter) in an *Rpv12^+^*/*Rpv12^−^ Rpv3^−^*/*Rpv3^−^* resistant parent (panels **A** and **C**) and in an *Rpv12^+^*/*Rpv12^−^ Rpv3^+^*/*Rpv3^−^* resistant parent (panels **B** and **D**). For the sake of simplicity, cosegregating markers are shown only once on the *x*-axis of the QTL plots.
(JPG)

Figure S6 Validation of the *Rpv12* genetic interval in progeny derived from the cross ‘Kunbaràt’ x ‘Sarfeher’.
(PDF)

Figure S7 Description of the *Rpv12* region in the reference genome, in the *Rpv12^+^* resistant haplotype, and diversity among grapevines: (panel **A**) recombination in custom-made populations; (panel **B**) variable sites between *Rpv12^+^* and *V. vinifera* haplotypes in six single-copy genes at symmetrical positions with respect to *Rpv12* (the analysis was extended to additional *Vitis* accessions in the least variable genes (panel **C**); plots of nucleotide diversity between sites comparing *Rpv12^+^* and *V. vinifera* haplotypes, and haplotype diversity in the set of haplotypes (panel **D**); physically assembled BAC clones of ‘Cabernet Sauvignon’ that cover the scaffold junction in the PN40024 sequence assembly in the middle of the *Rpv12* locus (panel **E**); sequence identity and hierarchical clustering between physically arrayed NB-LRR gene copies in the *Rpv12* locus (panel **F**).
(JPG)

Figure S8 Introgression of the *Rpv12^+^* region in grapevine varieties and the surrounding chromosomal landscape. Genetic (panel **A**) and physical (panels **D**,**H**) distance of mapped markers; density of exons, class I and II TEs, unclassified high-copy sequences, and low-copy DNA (panel **C**); density of NB-LRR genes (panels **E**,**I**) and their phylogenetic relationships (panels **M**–**P**); and genotypes at markers in 23 varieties (panel **L**) are given at the chromosomal scale (panels **D**,**H**).
(JPG)

Figure S9 DNA fingerprinting and kinship of six accessions claimed to belong to the first filial generation of *V. amurensis* x *V. vinifera* crosses.
(JPG)

## References

[1] Wan Y, Schwaninger H, Li D, Simon CJ, Wang Y, et al (2008) The eco-geographic distribution of wild grape germplasm in China. Vitis 47: 77–80.

[2] Wan Y, Schwaninger H, Li D, Simon CJ, Wang Y, et al (2008) A review of taxonomic research on Chinese wild grapes. Vitis 47: 81–88.

[3] Wang J, Yuxiang G, Yihong B (2004) Comparison of characteristics of *Vitis amurensis* Rupr. varieties. Journal of Northeast Forestry University 32: 29–31.

[4] Blasi P, Blanc S, Wiedemann-Merdinoglu S, Prado E, Rühl EH, et al (2011) Construction of a reference linkage map of *Vitis amurensis* and genetic mapping of Rpv8, a locus conferring resistance to grapevine downy mildew. Theor Appl Genet 123: 43–53. 21404060 10.1007/s00122-011-1565-0

[5] Wan Y, Schwaninger H, He P, Wang Y (2007) Comparison of resistance to powdery mildew and downy mildew in Chinese wild grapes. Vitis 46: 132–136.

[6] Loskutov IG (1999) Vavilov and his institute. A history of the world collection of plant genetic resources in Russia. IPGRI, Rome, Italy: 188 p.

[7] Fangmei L, Fengqin Z (1989) Grape breeding in China. In: Proceedings of the Fifth International Symposium on Grape Breeding, 212–215.

[8] Di Gaspero G, Copetti D, Coleman C, Castellarin SD, Eibach R, et al (2012) Selective sweep at the Rpv3 locus during grapevine breeding for downy mildew resistance. Theor Appl Genet 124: 277–286. 21947344 10.1007/s00122-011-1703-8

[9] Bellin D, Peressotti E, Merdinoglu D, Wiedemann-Merdinoglu S, Adam-Blondon AF, et al (2009) Resistance to *Plasmopara viticola* in grapevine ‘Bianca’ is controlled by a major dominant gene causing localised necrosis at the infection site. Theor Appl Genet 120: 163–176. 19821064 10.1007/s00122-009-1167-2

[10] Casagrande K, Falginella L, Castellarin SD, Testolin R, Di Gaspero G (2011) Defence responses in *Rpv3*-dependent resistance to grapevine downy mildew. Planta 234: 1097–1109. 21735199 10.1007/s00425-011-1461-5

[11] Koleda I (1975) Ergebnisse von Kreuzungen zwischen *Vitis amurensis* und *Vitis vinifera* in der Züchtung frostwiederstandfähiger Reben. Vitis 14: 1–5.

[12] Schwander F, Eibach R, Fechter I, Hausmann L, Zyprian E, et al (2012) Rpv10: a new locus from the Asian Vitis gene pool for pyramiding downy mildew resistance loci in grapevine. Theor Appl Genet 124: 163–176. 21935694 10.1007/s00122-011-1695-4

[13] Di Gaspero G, Cipriani G, Adam-Blondon A-F, Testolin R (2007) Linkage maps of grapevine displaying the chromosomal locations of 420 microsatellite markers and 82 markers for *R*-gene candidates. Theor Appl Genet 114: 1249–1263. 17380315 10.1007/s00122-007-0516-2

[14] Jaillon O, Aury J-M, Noel B, Policriti A, Clepet C, et al (2007) The grapevine genome sequence suggests ancestral hexaploidization in major angiosperm phyla. Nature 449: 463–468. 17721507 10.1038/nature06148

[15] Moroldo M, Paillard S, Marconi R, Legeai F, Canaguier A, et al (2008) A physical map of the heterozygous grapevine ‘Cabernet Sauvignon’ allows mapping candidate genes for disease resistance. BMC Plant Biol 8: 66. 18554400 10.1186/1471-2229-8-66 PMC2442077

[16] Merdinoglu D, Wiedemann-Merdinoglu S, Coste P, Dumas V, Haetty A, et al (2003) Genetic analysis of downy mildew resistance derived from *Muscadinia rotundifolia* . Acta Hort 603: 451–456.

[17] Cannon SB, Zhu H, Baumgarten AM, Spangler R, May G, et al (2002) Diversity, distribution, and ancient taxonomic relationships within the TIR and non-TIR NBS-LRR resistance gene subfamilies. J Mol Evol 54: 548–562. 11956693 10.1007/s0023901-0057-2

[18] Dick MW (2002) Towards an understanding of the evolution of the downy mildews. In: Spencer-Phillips PTN, Gisi U, Lebeda A (eds) Advances in downy mildew research, vol 1 . Kluwer, Dordrecht, pp. 1–59.

[19] Maddison WP, Knowles LL (2006) Inferring phylogeny despite incomplete lineage sorting. Syst Biol 55: 21–30. 16507521 10.1080/10635150500354928

[20] Zecca G, Abbott JR, Sun WB, Spada A, Sala F, et al (2012) The timing and the mode of evolution of wild grapes (*Vitis*). Mol Phylogenet Evol 62: 736–747. 22138159 10.1016/j.ympev.2011.11.015

[21] Bacilieri R, Lacombe T, Le Cunff L, Di Vecchi-Staraz M, Laucou V, et al (2013) Genetic structure in cultivated grapevines is linked to geography and human selection. BMC Plant Biol 13: 25. 23394135 10.1186/1471-2229-13-25 PMC3598926

[22] Myles S, Boyko AR, Owens CL, Brown PJ, Grassi F, et al (2011) Genetic structure and domestication history of the grape. Proc Natl Acad Sci USA 108: 3530–3535. 21245334 10.1073/pnas.1009363108 PMC3048109

