# Supplementary figures and images for: Historical Introgression of the Downy Mildew Resistance Gene Rpv12 from the Asian Species Vitis amurensis into Grapevine Varieties
Source: PLoS One. 2013 Apr 12;8(4):e61228. doi: 10.1371/journal.pone.0061228 (PMC3625174; doi:10.1371/journal.pone.0061228)

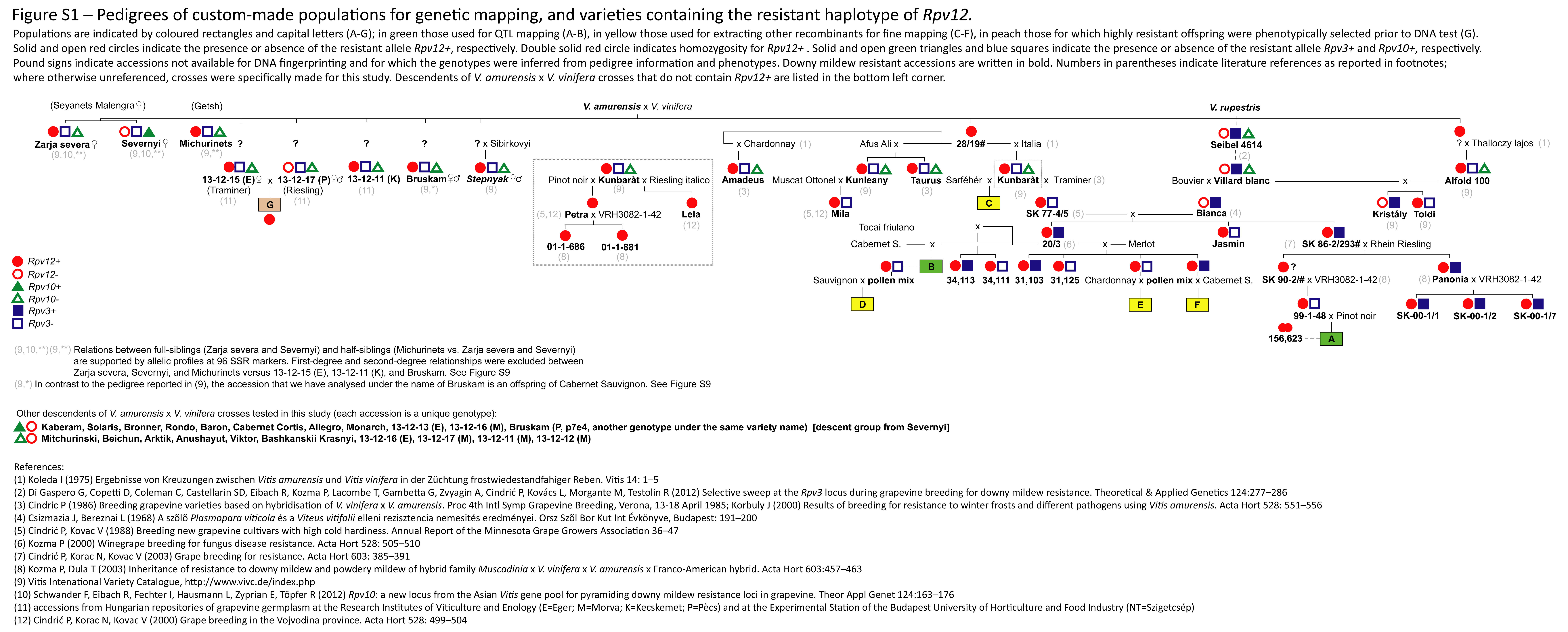

Supplement: Figure S1 — Pedigrees of custom-made populations for genetic mapping, and varieties containing the resistant haplotype of Rpv12. (JPG) [file pone.0061228.s001.jpg]

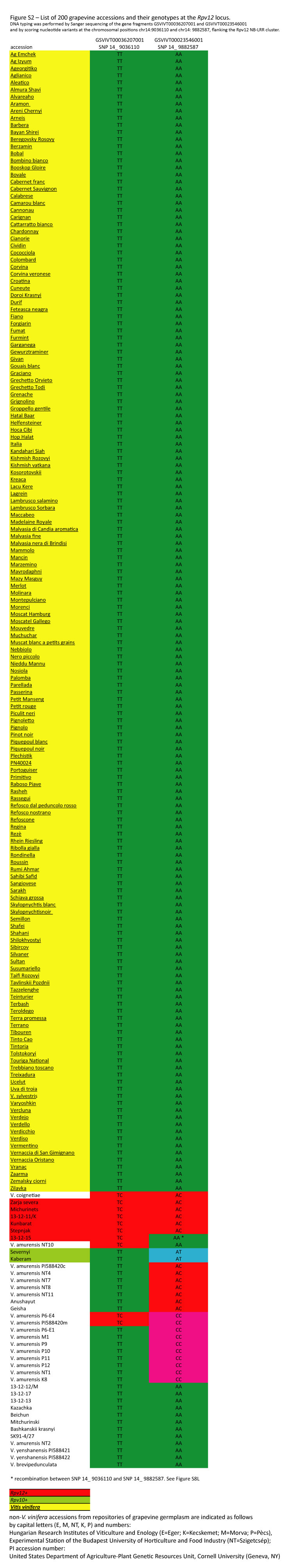

Supplement: Figure S2 — List of 200 grapevine accessions and their genotypes at the Rpv12 locus. DNA typing was performed by Sanger sequencing of the gene fragments GSVIVT00036207001 and GSVIVT00023546001 and by scoring nucleotide variants at the chromosomal positions chr14:9036110 and chr14: 9882587, flanking the Rpv12 NB-LRR cluster. (JPG) [file pone.0061228.s002.jpg]

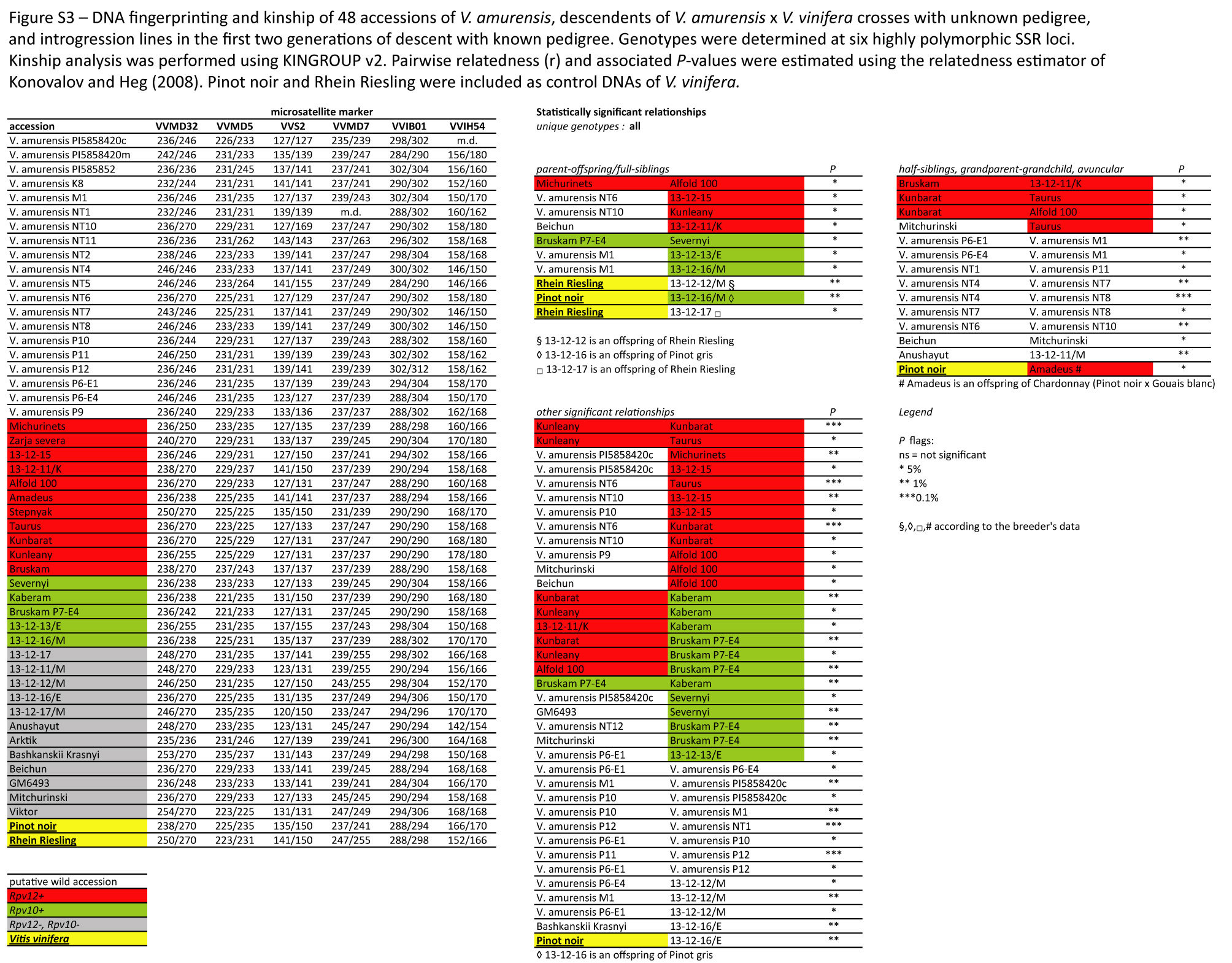

Supplement: Figure S3 — DNA fingerprinting and kinship of 48 accessions of V. amurensis, descendents of V. amurensis x V. vinifera crosses with unknown pedigree, and introgression lines in the first two generations of descent with known pedigree. (JPG) [file pone.0061228.s003.jpg]

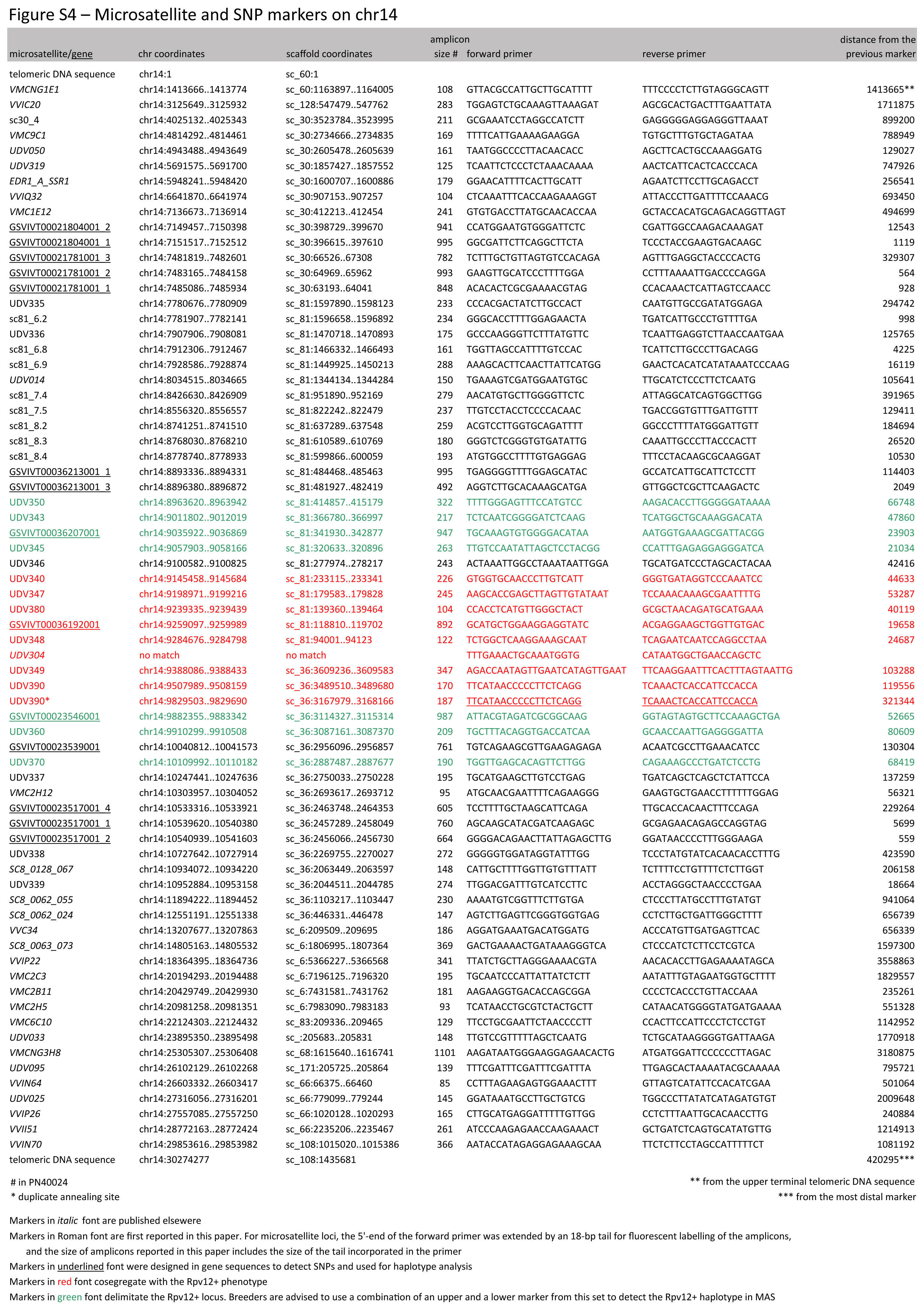

Supplement: Figure S4 — Microsatellite and SNP markers on chr14. (JPG) [file pone.0061228.s004.jpg]

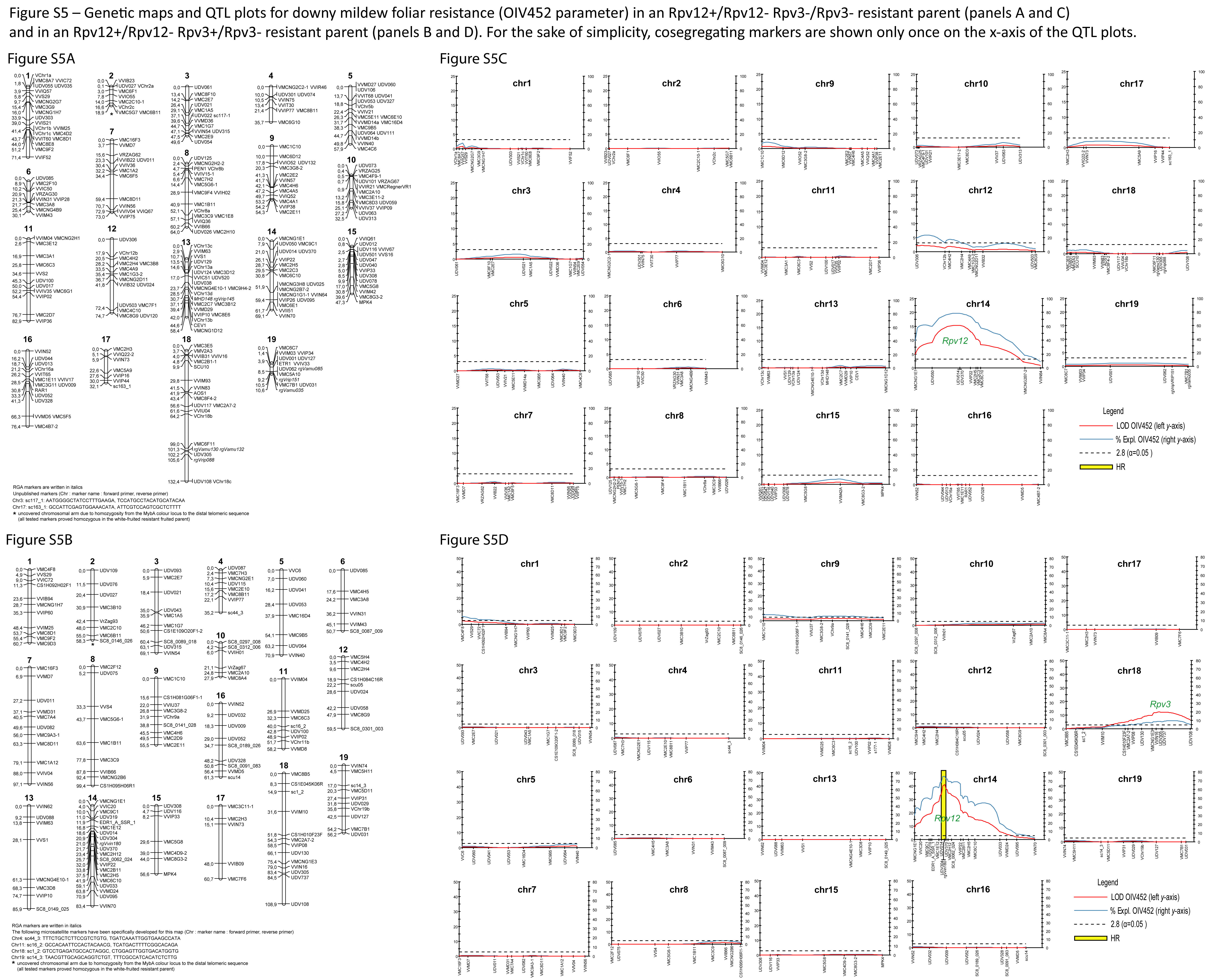

Supplement: Figure S5 — Genetic maps and QTL plots for downy mildew foliar resistance (OIV452 parameter) in an Rpv12+/Rpv12− Rpv3−/Rpv3− resistant parent (panels A and C) and in an Rpv12+/Rpv12− Rpv3+/Rpv3− resistant parent (panels B and D). For the sake of simplicity, cosegregating markers are shown only once on the x-axis of the QTL plots. (JPG) [file pone.0061228.s005.jpg]

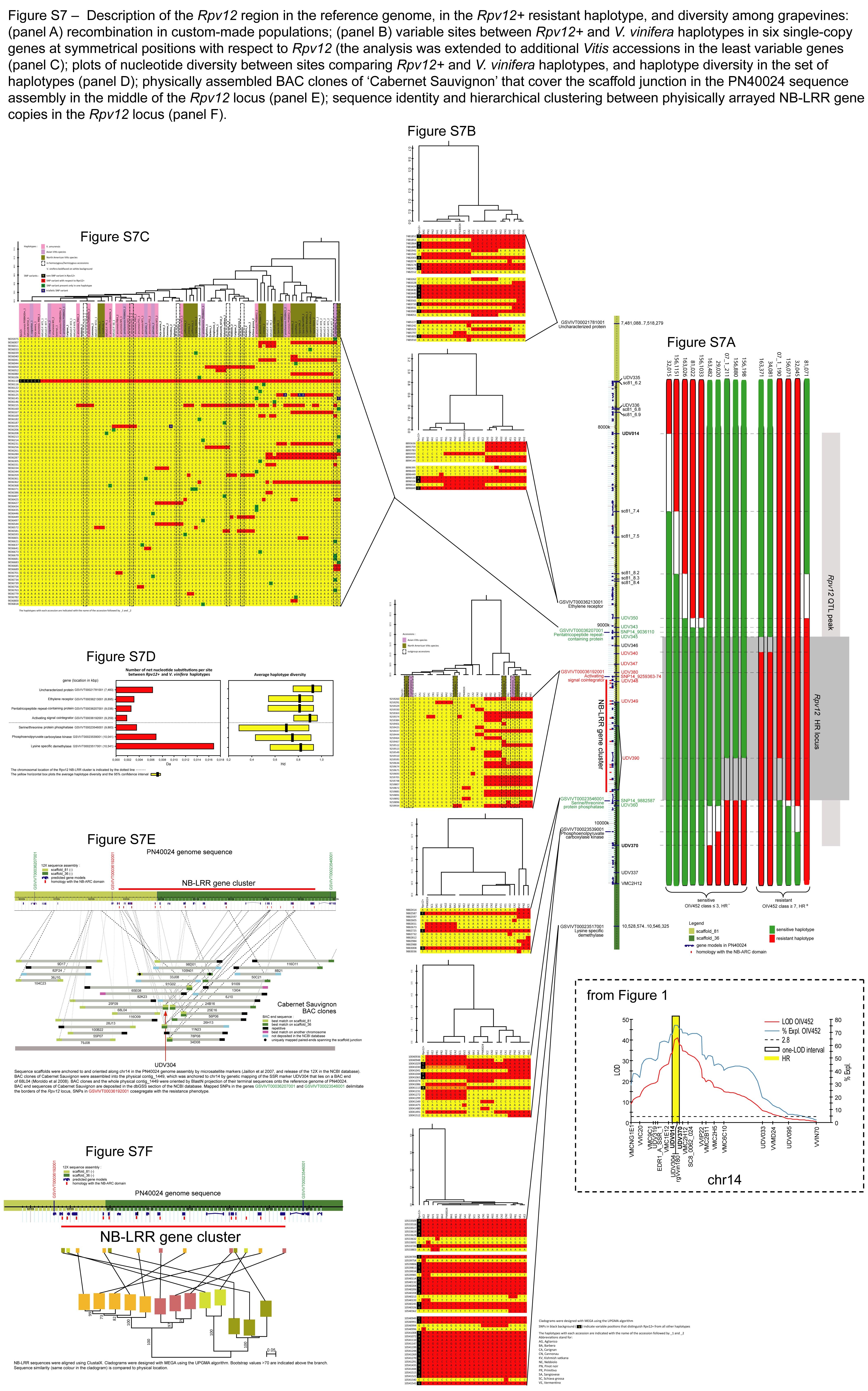

Supplement: Figure S7 — Description of the Rpv12 region in the reference genome, in the Rpv12+ resistant haplotype, and diversity among grapevines: (panel A) recombination in custom-made populations; (panel B) variable sites between Rpv12+ and V. vinifera haplotypes in six single-copy genes at symmetrical positions with respect to Rpv12 (the analysis was extended to additional Vitis accessions in the least variable genes (panel C); plots of nucleotide diversity between sites comparing Rpv12+ and V. vinifera haplotypes, and haplotype diversity in the set of haplotypes (panel D); physically assembled BAC clones of ‘Cabernet Sauvignon’ that cover the scaffold junction in the PN40024 sequence assembly in the middle of the Rpv12 locus (panel E); sequence identity and hierarchical clustering between physically arrayed NB-LRR gene copies in the Rpv12 locus (panel F). (JPG) [file pone.0061228.s007.jpg]

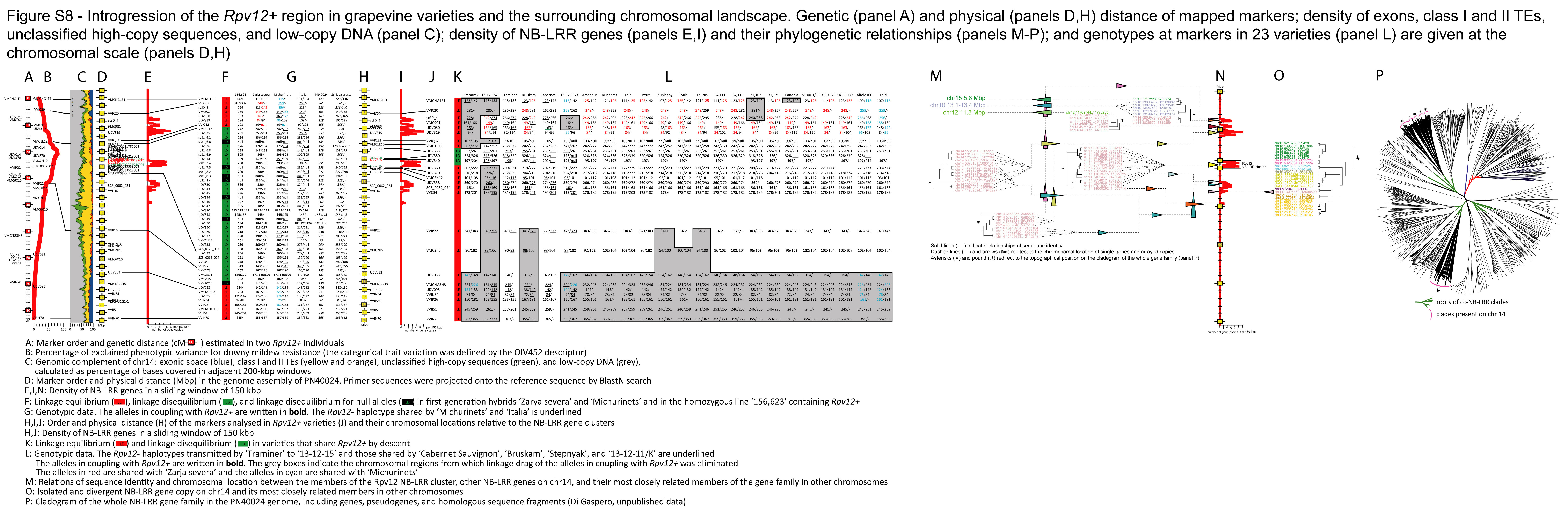

Supplement: Figure S8 — Introgression of the Rpv12+ region in grapevine varieties and the surrounding chromosomal landscape. Genetic (panel A) and physical (panels D,H) distance of mapped markers; density of exons, class I and II TEs, unclassified high-copy sequences, and low-copy DNA (panel C); density of NB-LRR genes (panels E,I) and their phylogenetic relationships (panels M–P); and genotypes at markers in 23 varieties (panel L) are given at the chromosomal scale (panels D,H). (JPG) [file pone.0061228.s008.jpg]

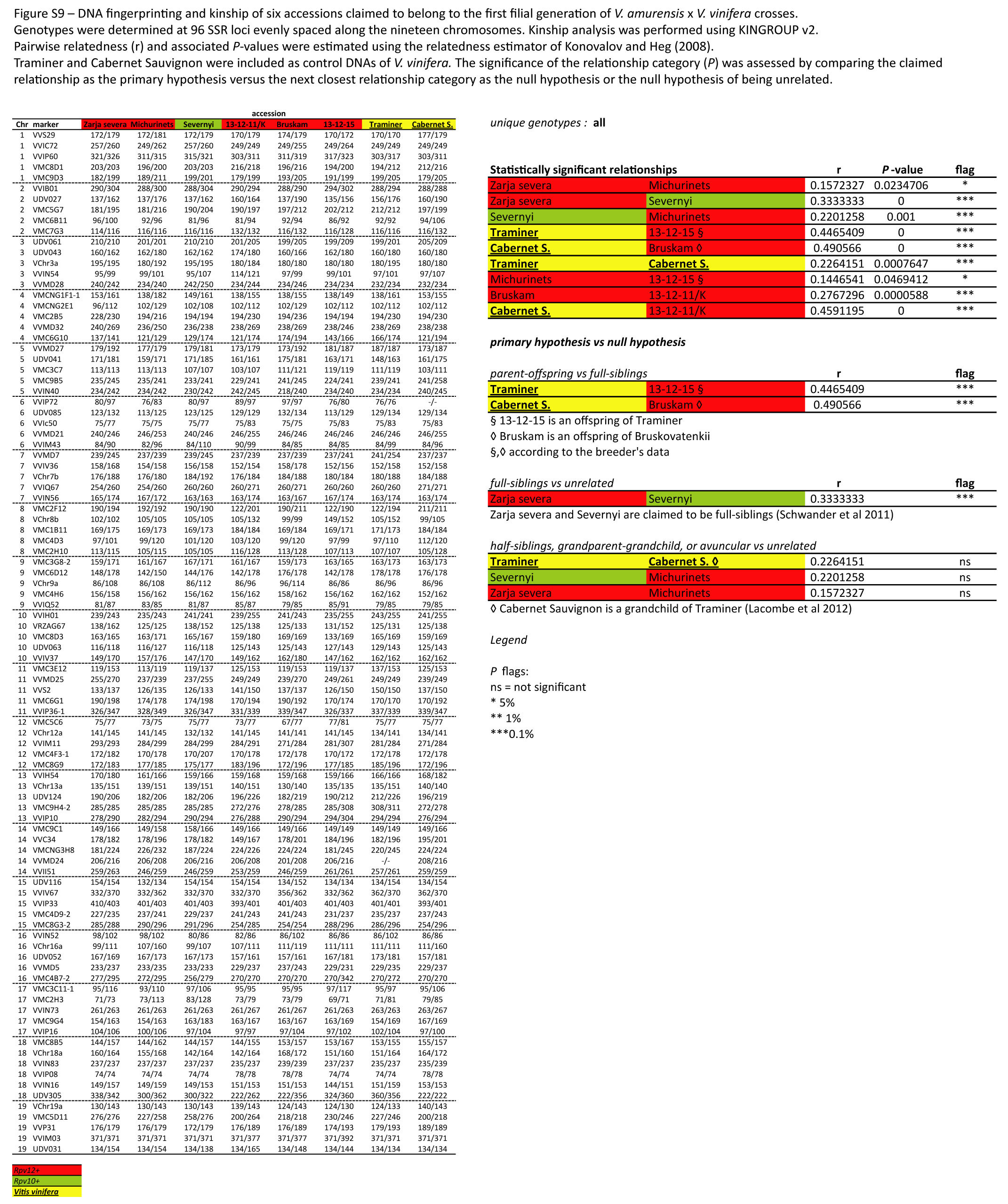

Supplement: Figure S9 — DNA fingerprinting and kinship of six accessions claimed to belong to the first filial generation of V. amurensis x V. vinifera crosses. (JPG) [file pone.0061228.s009.jpg]
