# Supplementary material for: Historical Introgression of the Downy Mildew Resistance Gene Rpv12 from the Asian Species Vitis amurensis into Grapevine Varieties
Source: PLoS One. 2013 Apr 12;8(4):e61228. doi: 10.1371/journal.pone.0061228 (PMC3625174; doi:10.1371/journal.pone.0061228)
Supplement: Figure S6 — Validation of the Rpv12 genetic interval in progeny derived from the cross ‘Kunbaràt’ x ‘Sarfeher’. (PDF) [file pone.0061228.s006.pdf]

Figure S6 – Validation of the *Rpv12* genetic interval in progeny derived from the cross 'Kunbaràt' x 'Sarfeher'. Phenotyping for downy mildew resistance was performed in open field using 185 seedlings. Resistance was scored during two consecutive seasons using the OIV452 descriptor. The progeny displayed two phenotypic classes (OIV452 class 3 = sensitive, absence of HR; OIV452 class 7 = resistant, presence of HR) and, according to this parameter, the trait segregated in a Mendelian fashion (χ^2 = 0.53; d.f. 1). Genotyping of all seedlings with the *Rpv12* flanking markers UDV014 and UDV370 confirmed linkage with the trait. Two recombinants excluded the possibility that the causal gene is located upstream of UDV014 or downstream of UDV370.

Observed phenotypic classes for the OIV452 parameter on the abaxial surface of the whole leaf (above) and in the intercoastal fields of the lamina infected by the pathogen (below).

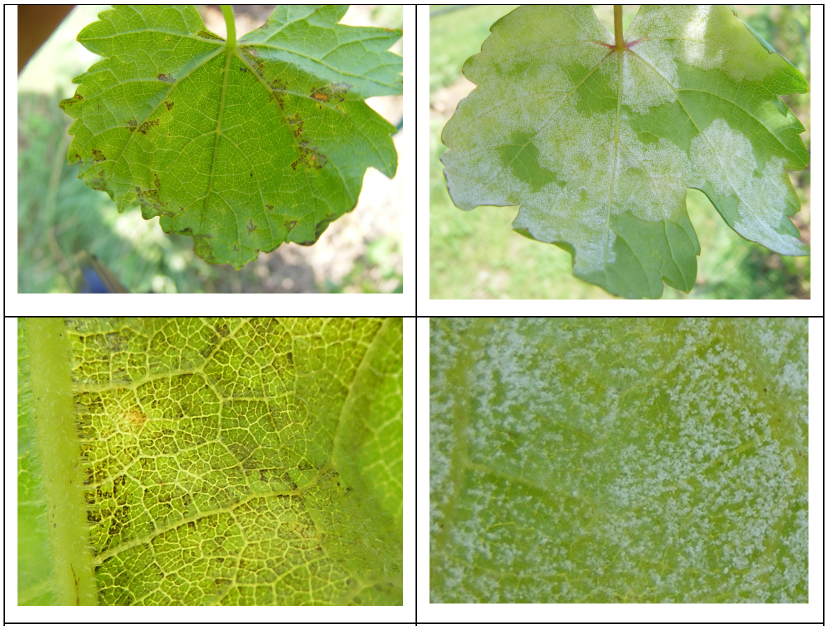

Class 7	Class 3

Genotypes and phenotypes in 185 offspring of 'Kunbaràt'. The red portions of the diagram indicate the resistant haplotype donated by 'Kunbaràt' and identified by the alleles 159 and 218 (bp) at the markers UDV014 and UDV370. The white portions of the diagram indicate the susceptible haplotype donated by 'Kunbaràt' carrying the alleles 149 and 214 (bp) at the markers UDV014 and UDV370.

n. of seedlings	98	85	1	1
genotype	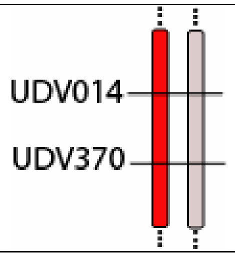	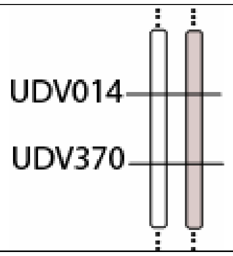	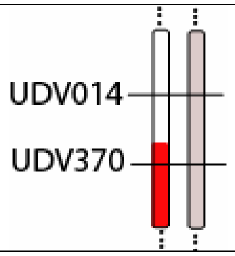	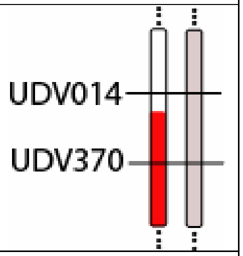
phenotype	class 7	class 3	class 3	class 7
